# Case Report: Refractory hidradenitis suppurativa complicated by IgA vasculitis with nephritis: successful long-term control with surgery and bimekizumab

**DOI:** 10.3389/fimmu.2025.1721038

**Published:** 2026-01-06

**Authors:** Ayaka Yasuda, Natsuko Sasaki, Emi Hasegawa, Yu Sawada

**Affiliations:** 1 Department of Dermatology, University of Occupational and Environmental Health, Kitakyushu, Japan; 2 Department of Cardiovascular, University of Occupational and Environmental Health, Kitakyushu, Japan

**Keywords:** hidradenitis suppurativa, bimekizumab, IL-17, case report, IgA vasculitis

## Abstract

Hidradenitis suppurativa (HS) is a chronic inflammatory skin disease that can rarely be complicated by systemic immune phenomena. We report a 41-year-old woman with severe HS refractory to adalimumab who developed fever, palpable purpura, and renal involvement. Skin biopsy confirmed IgA leukocytoclastic vasculitis, and renal biopsy revealed IgA nephritis. She was treated with corticosteroids, rituximab, and surgery, but proteinuria persisted. Initiation of bimekizumab led to marked improvement of HS lesions and a significant reduction in proteinuria. Over more than one year of follow-up, she achieved sustained HS control without recurrence of vasculitis or treatment-related adverse events. This case highlights the potential role of dual IL-17A/F inhibition in managing refractory HS complicated by IgA vasculitis and nephritis.

## Introduction

Hidradenitis suppurativa (HS) is a chronic inflammatory skin disease characterized by painful nodules, abscesses, and sinus tracts in intertriginous areas (1). Severe cases are refractory to conventional treatments and may require biologic therapy or surgery (2). Severe HS is associated with systemic inflammation and immune dysregulation. In this context, IgA vasculitis, an immune complex–mediated small vessel vasculitis, manifests with purpura, arthritis, gastrointestinal symptoms, and renal involvement (3, 4). Although the coexistence of HS and IgA vasculitis is exceedingly rare, previous reports have implicated biologic agents such as tumor necrosis factor (TNF)-α inhibitors, including adalimumab (5). These observations raise important questions regarding management strategies in HS complicated by systemic immune phenomena.

Several immunologic mechanisms may underlie the association between HS and vasculitis. Both diseases share a predominance of neutrophil-mediated inflammation and dysregulation of cytokine pathways involving IL-1β, IL-6, IL-17, and TNF-α (8, 9). Persistent neutrophil activation in HS can promote immune complex formation and vascular inflammation, potentially triggering IgA vasculitis in susceptible patients. These overlapping inflammatory pathways provide a plausible explanation for the coexistence of HS and IgA vasculitis and support the therapeutic rationale for IL-17 inhibition in such cases.

Herein, we describe a patient with refractory HS complicated by IgA vasculitis and nephritis, whose disease was successfully controlled with surgery and bimekizumab treatment.

## Case presentation

A 41-year-old female with no prior medical history other than epilepsy presented with a 10-year history of HS involving both axillae, groins, and buttocks. At initial evaluation, her disease was classified as Hurley stage III with an IHS4 score of 68. Her HS remained active even in the treatment of adalimumab. One year before referral, she experienced high fever, was unable to take her antiepileptic medication and suffered epileptic seizures requiring emergency hospitalization.

**Figure 1:**
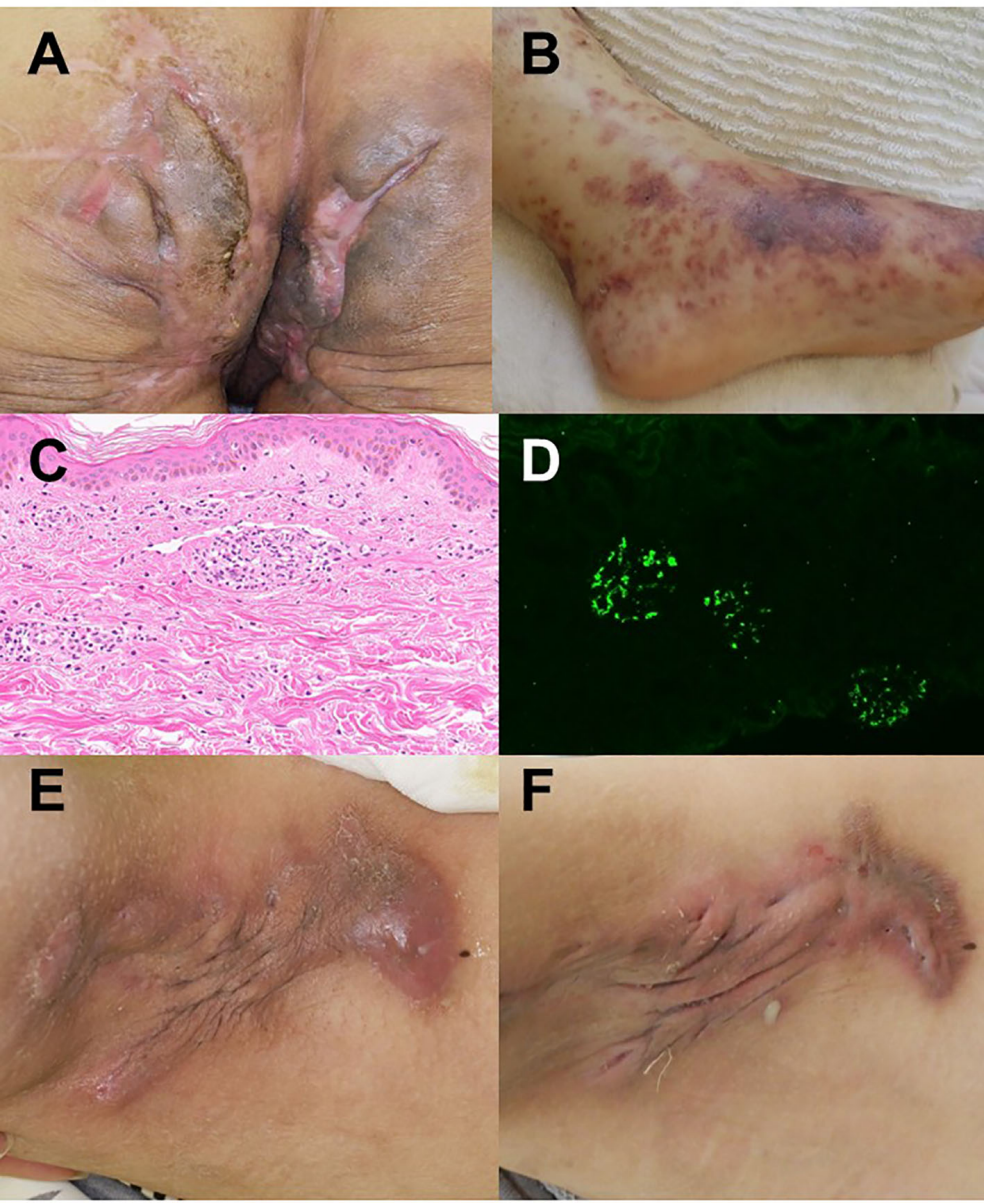
Clinical and pathological findings. **(A)** Recurrent buttock lesions with erythematous abscesses and draining fistulas. **(B)** Palpable purpura with edema on both lower legs appearing simultaneously with buttock flare. **(C)** Skin biopsy of purpuric lesions showing leukocytoclastic vasculitis with perivascular neutrophilic infiltration. **(D)** Renal biopsy confirming IgA nephritis, showing mesangial proliferation and IgA deposition. **(E)** Axillary lesions before bimekizumab treatment showing multiple inflammatory nodules, abscess formation, and sinus tracts. **(F)** Six months after bimekizumab initiation, the lesions showed marked regression with minimal residual inflammation and slight pus drainage.

She was referred to our hospital for her intractable HS condition required surgical treatment. Under general anesthesia, wide excision of both inguinal regions was performed, which eliminated fistulas in those areas and resulted in sustained local control. However, six fistulas and three abscesses persisted in the buttocks. During follow-up, she continued developing skin inflammation in the buttock lesions with high fever (**Figure 1A**). On the same day, palpable purpura appeared on both lower legs with edema (**Figure 1B**).

Laboratory findings showed elevated serum IgA and urinary abnormalities with proteinuria and hematuria. Skin biopsy of purpuric lesions demonstrated leukocytoclastic vasculitis (**Figure 1C**), and renal biopsy confirmed IgA nephritis (**Figure 1D**).

She was treated with systemic corticosteroids and rituximab and also underwent surgical treatment for buttock fistulas. Despite these interventions, proteinuria persisted. Subsequently, bimekizumab was initiated for HS, which resulted not only in dramatic improvement of cutaneous lesions but also in significant reduction of proteinuria. Laboratory data before bimekizumab treatment showed elevated inflammatory markers and renal impairment (BUN 38.24 mg/dL, creatinine 1.27 mg/dL, eGFR 38.29 mL/min/1.73 m²). Six months after treatment initiation, these values improved markedly (BUN 18.0 mg/dL, creatinine 0.63 mg/dL, eGFR 81.87 mL/min/1.73 m²), indicating recovery of renal function along with clinical improvement of both hidradenitis suppurativa and IgA vasculitis (**Figures 1E, F**). Over more than one year of follow-up, her HS activity markedly improved without recurrence of IgA vasculitis was observed.

## Discussion

HS is increasingly recognized as a systemic inflammatory disorder with potential associations beyond cutaneous manifestations. Our patient represents a rare case of refractory HS complicated by IgA vasculitis with nephritis. We summary previous report of HS complication with vasculitis (5–7). To our knowledge, very few cases of HS complicated by vasculitis have been documented, and none have clearly described successful long-term control using IL-17A/F inhibition.

The coexistence of HS and IgA vasculitis raises several mechanistic considerations. Chronic HS is characterized by persistent neutrophil-dominated inflammation and dysregulated cytokine pathways, particularly involving TNF-α, IL-1β, IL-23, and IL-17 (8). These immune alterations may predispose to secondary immune complex–mediated conditions such as IgA vasculitis (9). In our case, systemic inflammation from severe HS combined with secondary bacterial infection likely acted as a trigger for vasculitis onset.

Another potential contributor is anti-TNF therapy. Several case reports and systematic reviews have implicated TNF-α inhibitors, including adalimumab, in the development of leukocytoclastic vasculitis and IgA vasculitis (10). Proposed mechanisms include immune complex deposition, and a paradoxical shift toward autoimmunity. In most reported cases, vasculitis improved after discontinuation of the offending biologic. Thus, in patients with HS who develop vasculitis during anti-TNF therapy, prompt recognition and discontinuation of the drug are essential.

Our patient’s disease control was ultimately achieved by a dual approach. First, wide excision and deroofing surgery treatment was critical for eliminating chronic sinus tracts that serve as reservoirs of inflammation and infection. Second, bimekizumab provided effective suppression of HS activity. By simultaneously neutralizing IL-17A and IL-17F, bimekizumab may offer broader control of neutrophil-driven inflammation compared with IL-17A–specific inhibitors. Importantly, IL-17 inhibitors have not been strongly associated with immune complex–mediated vasculitis, in contrast to TNF blockades. This pharmacological distinction may explain the absence of vasculitis relapse during more than one year of follow-up.

## Conclusion

In summary, we report a rare case of refractory HS complicated by IgA vasculitis with nephritis. Our patient achieved long-term control through a combined approach of surgical excision and bimekizumab therapy. The success of this strategy underscores the central role of the IL-23/IL-17 axis in HS pathogenesis and highlights the potential advantages of IL-17A/F blockade.

## Data Availability

The original contributions presented in the study are included in the article/supplementary material. Further inquiries can be directed to the corresponding author.
